# Effects of Orthokin, Sensikin and Persica mouth rinses on the force degradation of elastic chains and NiTi coil springs

**DOI:** 10.15171/joddd.2016.016

**Published:** 2016-06-15

**Authors:** Zahra Javanmardi, Parisa Salehi

**Affiliations:** ^1^Post graduate student, Department of Orthodontics, Orthodontic Research Center, School of Dentistry, Shiraz University of Medical Sciences, Qom Abad Street, Shiraz, Iran; ^2^Professor, Department of Orthodontics, Orthodontic Research Center, School of Dentistry, Shiraz University of Medical Sciences, Qom Abad Street, Shiraz, Iran

**Keywords:** Elastomeric chain, force degradation, mouth rinse, NiTi coil spring

## Abstract

***Background.*** Elastomeric chains and NiTi coil springs are two major traction aids in orthodontic tooth movements. Force degradation occurs over time in both groups, with higher percentages in elastic chains. The effects of environmental factors and some mouth rinses on this force decay have been previously studied. No study has been performed to evaluate the effect of current popular mouth rinses such as Orthokin, Sensikin and Persica on this force degradation.

***Methods***. Forty pieces of elastic chains consisting of 5 loops (Ortho Technology, USA) and 40 NiTi closed coil springs (3M Unitek, Germany) were divided into 4 groups: control (artificial saliva), Orthokin mouthwash, Sensikin mouthwash and Persica mouthwash. All the groups were kept in an incubator at 37°C for 3 weeks. In the test groups, the samples were immersed in mouthwash twice a day. Force degradation was measured at 5 time intervals: baseline, 1 hour, 24 hours, 1 week and 3 weeks, using a digital force gauge. Repeated-measures ANOVA and one-way ANOVA were used for statistical analysis.

***Results.*** Force decay occurred over time in both elastic chainand coil spring groups. In elastic chain group, after 3 weeks, Orthokin mouth rinse had significantly lower force degradation compared to other groups (P < 0.05) and in coil spring group there were no statistically significant differences in force degradation after 3 weeks between the subgroups (P > 0.05).

***Conclusion.*** Based the results of this study, these three mouthwashes did not increase the force degradation of orthodontic traction aids under study.

## Introduction

Elastomeric chains are used in orthodontic tooth movements for different purposes, including midline correction, space closure and moving the impacted teeth.^1^They are broadly employed because they are hygienic, cost-effective and easy to use.^2^

Elastomeric chains are made of polyurethane and because of their viscoelastic properties, they lose their force over time;^3^therefore,different studies have been carried out to show this force decay.^1,4-7^ During the first day of use the force loss is maximal; then the force decay continues to decrease at a much consistent rate.^8^ Some factors can affect this force loss such as temperature^9^ and pH changes.^10^

Nickel-titanium (NiTi) coil springs are another orthodontic traction aids and according to previous studies^11-13^ they are preferred for space closure compared to elastomeric chains since their forces are light and continuous; therefore, they close spaces more consistently than elastomeric chains. In addition, environmental factors like humidity and pH variations exert minor effects on NiTi coil springs.^14^

The effects of water, Coke, turmeric solution and temperature on elastic chains and NiTi coil springs have been studied, leading to the conclusion that elastomeric chains were affected by all the test environments while NiTi springs were only affected by temperature.^14^

The force decay of elastomeric chains and stainless steel and NiTi coil springs were compared under dry conditions and in artificial saliva and a mouth rinse of chlorhexidine and NaF. It was concluded that NiTi coil springs exhibited the minimum percentage of force degradation under all the conditions and elastomeric chains exhibited the highest percentage.^11^

Today, the use of mouth rinses is on the rise for better oral hygiene, especially among orthodontic patients.^15^The effect of different mouth rinses such as alcohol,^3^ fluoride^16^ and bleaching agents containing mouth rinses^17^ on the force decay of elastomeric chains were studied. These studies concluded that alcohol caused an increase in force decay of elastomeric chains over time,^3^ but the bleaching agent had no effect on force decay of elastomeric chains^17^ and NaF mouth rinse did not affect the force decay of elastomeric chains with conventional orthodontic force ranges.^16^

Orthokin and Sensikin mouthwashes are prescribed by many orthodontists. According to the product’s brochure, Orthokin mouth rinse contains sodium fluoride, chlorhexidine digluconate and zinc acetate, and can be used for caries prevention and bacterial control. Sensikin mouth rinse has potassium nitrate and sodium fluoride, which can be used as a mouth rinse to prevent caries and hypersensitivity.Persica is a native herbal mouth rinse with Miswak herb extract as its main component.^18^Some previous studies have shown that this herbal medicine or its extract can reduce microbial plaque and gingival bleeding.^19-21^ As Persica mouth rinse results in less tooth discoloration and unpleasant taste compared to chlorhexidine^18^ and because of its low cost, it might be a good substitute for chlorhexidine-containing mouth rinses.

There is no information available on the effect of Orthokin, Sensikin and Persica mouth rinses on the force degradation of elastomeric chains and NiTi coil springs; therefore, the aim of this study was to evaluate the effects of these three mouthwashes on the force decay of elastomeric chains and NiTi coil springs.

## Methods

This study did not involve the use of any animals or human data or tissues, and thus, an ethics approval was not required.

In this laboratory study, forty continuous clear elastomeric chain specimens (Ortho Technology, USA) each consisting of 5 loops^5^ and forty NiTi coil springs (3M Unitek, Germany) measuring 12 mm in length^11,12,22^ were randomly divided into 4 groups:

Group 1: Artificial saliva

Group 2: Persica mouth rinse

Group 3: Orthokin mouth rinse

Group 4: Sensikin mouth rinse

**Figure 1:**
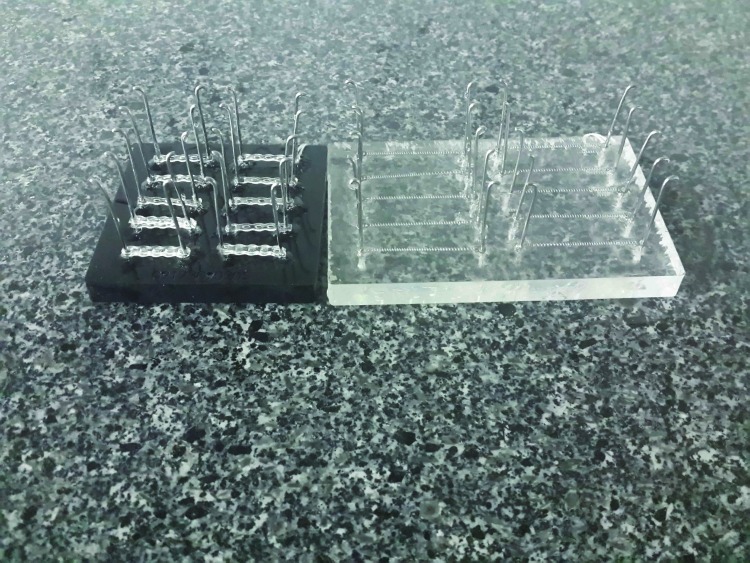
Plastic blocks with stainless steel pins.

Eight plastic blocks (Figure 1) with 10 pairs of stainless steel pins, to maintain each sample at its specific length, were prepared to carry samples without relaxation.^11^

The distance between the pins was 15.5 mm for maintaining elastomeric chains. This distance was measured by stretching 5 specimens to produce 200±5 g of force.^11^

The pins for NiTi springs were 33 mm apart based on the manufacturer’s instructions.

The length of the specimens was considered as being constant because the mean rate of space closure (0.19‒0.24 mm per week) is negligible.^23^

In the case of artificial saliva, the samples were immersed in artificial saliva solution and stored in an incubator at 37°C for 3 weeks.

In the case of mouth rinses, the samples were initially incubated in artificial saliva solution at 37°C in an incubator. After that, the blocks were retrieved from artificial saliva and immersed in mouthwash solution in glass containers for 1 minute twice daily. These two daily exposures were separated by 9 hours^3^ for three weeks. At the end of the immersion period, the blocks were immersed in another saliva container, specific for each mouth rinse for 30 minutes to mimic the use of the mouthwash by the patient. Then they were rinsed in water to prevent the entrance of the mouth rinse into the main saliva container, and again were returned to the saliva container at 37°C.

Measurement of the force generated by each sample was performed by means of a digital force gauge (Force Gauge Lutron/Model (FG-5020)/Accuracy: ±(0.5% + 2 digits)/measuring capacity: 20.00 kg) at five time intervals:^11^

1: baseline (0 hour) just before incubation

2: 1-hour interval

3: 24-hour interval

4: one-week interval

5: three-week interval

**Figure 2:**
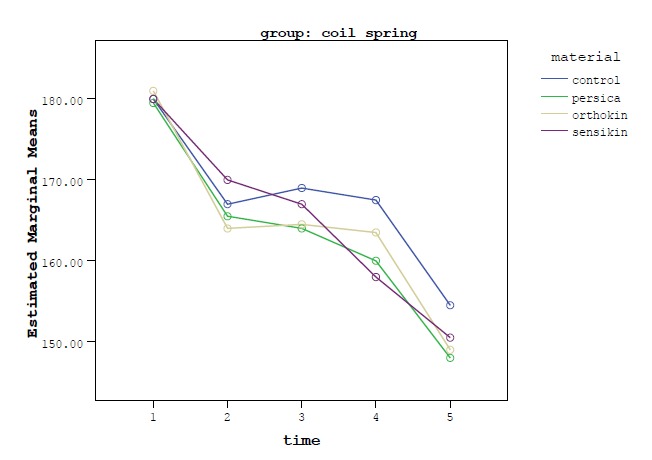
Digital force gauge and its stand.

The force gauge was kept in its specific stand to perform all the measurements at identical horizontal and vertical positions (Figure 2). In order to transfer the samples without any change in their length, plastic blocks were taken close to the gauge which had been adjusted to the specific length; then each side of the samples was separated from the pin and immediately connected to the hook of the gauge.

Force degradation was obtained by the following equation:

“%FD= 100 × (IF - FT)/IF” ^11,24^

(FD: force degradation, IF: initial force, FT: force at specific time interval).

## Results

**Figure 3:**
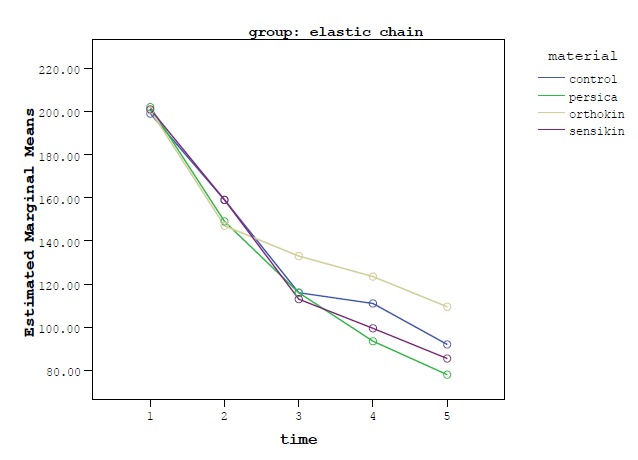
Graphic representation of coil spring force (left) and elastic chain force (right) levels over the 3-week experimental period for all the experimental groups. Times 1, 2, 3, 4 and 5 indicate initial, 1 hour, 24 hours, 1 week and 3 weeks, respectively.

Two-sample repeated measures (RM) ANOVA was used to assess the effect of materials over time. One-sample RM ANOVA/Sidak test (intra-group comparison) was employed for subgroup analyses. One-way ANOVA/Tukey tests were used to compare percentages of force decay after 3 weeks between experimental materials. SPSS 18.0 (Chicago, IL, USA) was used for data analysis. Statistical significance was set at P<0.05. Figures 3 show force levels of NiTi coil springs and elastic chains over time in all the experimental groups.

The intra-group analysis is summarized in Tables 1 and 2.

**Table 1:** Comparison of coil spring mean force values (g) ± standard deviations (SD) within each group at different evaluation intervals

Time	Control		Persica		Orthokin		Sensikin	
	Mean (SD)	P-value	Mean (SD)	P-value	Mean (SD)	P-value	Mean (SD)	P-value
Baseline	180(4.08)	1h	179.5(3.68)	1h	181(3.94)	1h	180(3.33)	1h
		p=.003^*^		p=.001^*^		p=.001^*^		p=.047^*^
		24h p=.005^*^		24h p=.001^*^		24h p=.000^*^		24h p=.000^*^
		1w		1w		1w		1w
		p=.002^*^		p=.000^*^		p=.000^*^		p=.000^*^
		3w		3w		3w		3w
		p=.000^*^		p=.000^*^		p=.000^*^		p=.000^*^
1 h	167(5.37)	24h	165.5(4.97)	24h	164(6.58)	24h	170(7.81)	24h
		p=.996		p=.999		p=1.000		p=0.970
		1w		1w		1w		1w
		p=1.000		p=.110		p=1.000		p=.016^*^
		3w		3w		3w		3w
		p=.002^*^		p=.001^*^		p=.006^*^		p=.000^*^
24 h	169(6.58)	1w	164(6.14)	1w	164.5(5.98)	1w	167(4.83)	1w
		p=1.000		p=.809		p=1.000		p=.050^*^
		3w		3w		3w		3w
		p=.002^*^		p=.004^*^		p=.000^*^		p=.000^*^
1 w	167.5(3.53)	3w	160(5.27)	3w	163.5(4.74)	3w	158(5.37)	3w*
		p=.001^*^		p=.007^*^		p=.003^*^		p=.017^*^
3w	154.5(5.50)		148(5.86)		149(5.67)		150.5(4.37)	

^*^statistically significant differences (P < 0.05) h: hour; w: week.

**Table 2:** Comparison of elastic chain mean force values (g) ± standard deviations (SD) within each group at different evaluation intervals

Time	Control		Persica		Orthokin		Sensikin	
	Mean (SD)	P-value	Mean (SD)	P-value	Mean (SD)	P-value	Mean (SD)	P-value
Baseline	199(3.94)	1h	202(3.49	1h	200.5(4.37)	1h	201(3.94))	1h
		p=.000^*^		p=.000^*^		p=.000^*^		p=.000^*^
		24h		24h		24h		24h
		p=.000^*^		p=.000^*^		p=.000^*^		p=.000^*^
		1w		1w		1w		1w
		p=.000^*^		p=.000^*^		p=.000^*^		p=.000^*^
		p=.000^*^		3w		3w		3w
1 h	159(7.74)	24h	149(12.42)	24h	147(11.83	24h	159(6.99)	24h
		p=.001^*^		p=.001^*^		p=.043^*^		p=.000^*^
		1w		1w		1w		1w
		p=.001^*^		p=.000^*^		p=.006^*^		p=.000^*^
		3w		3w		3w		3w
		p=.000^*^		p=.000^*^		p=.000^*^		p=.000^*^
**24 h**	116(15.42)	1w	116(11.00)	1w	133(9.48)	1w	113(9.18)	1w
		p=.988		p=.009^*^		p=.377		p=.005^*^
		3w		3w		3w		3w
		p=.083		p=.000^*^		p=.051		p=.001^*^
**1 w**	111(19.11)	3w	93.5(11.55)	3w	123.5(10.28)	3w	99.5(15.17)	3w
		p=.524		p=.198		p=.258		p=.113
3w	92(15.67)		78(11.83)		109.5(13.83)		85.5(11.16)	

^*^statistically significant differences (P <0 .05) h: hour; w: week.

For coil spring group in all the media force decay occurred over time, but a slight increase in force production occurred in the control group between 1- and 24-hour intervals and also between 1-hour and 1-week intervals, and in the Orthokin group between 1-hour and 24-hour intervals, which were not statistically significant (1).

According to 1, there was significant force degradation between the baseline and all the other time intervals. In the control, Persica and Orthokin groups, significant differences were found between 3-week and 1-hour, 3-week and 24-hour, and also 3-week and 1-week intervals, while in the Sensikin group all the comparisons were statistically significant except between 1- and 24-hour intervals.

The same test for elastic chain group showed force degradation over time in all the media. Statistically significant differences were found between baseline and other time intervals and also between 1-hour and other time intervals in the control and Orthokin groups. In the Persica and Sensikin mouth rinses all the comparisons were significantly different except between 1- and 3-week intervals (2).

3 shows analysis of the percentages of force degradation in all the media in the elastic chain group at the end of 3 weeks. Tukey test indicated that after 3 weeks, Orthokin mouth rinse resulted in significantly lower force degradation compared to other groups.

**Table 3:** Inter-group comparisons (Tukey test) of the percentages of force degradation for the elastic chain group at the end of the 3-week experimental period

**Material**	**Mean ±SD (force degradation %)**	**P-value**
**Control**	53.75 ± 7.91	Persica 0.055
		Orthokin 0.032^*^
		Sensikin 0.571 ^*^
**Persica**	61.39± 5.77	Orthokin 0.000^*^
		Sensikin 0.537^*^
**Orthokin**	45.42± 6.53	Sensikin 0.001^*^
**Sensikin**	57.49± 5.26	

Tables 4 and 5 show the same analysis for the coil spring group, indicating no statistically significant differences in force degradation after 3 weeks between the groups.

**Table 4:** Descriptive statistics of the percentages of force degradation in all the experimental media at the end of the 3-week period for the coil spring group

**Media**	**Number**	**Mean ± SD (FD %)**
**Control**	10	14.09± 4.36
**Persica**	10	17.51± 3.70
**Orthokin**	10	17.63± 3.73
**Sensikin**	10	16.35± 3.16

FD: Force degradation; SD: Standard deviation.

**Table 5:** One-way ANOVA of the percentages of force degradation after 3 weeks for the coil spring group

**Test**	**Sum of Suares**	**Df**	**Mean Square**	**F**	**Sig.**
**Between groups**	**80.909**	**3**	**26.970**	**1.900**	**.147**
**Within groups**	**510.986**	**36**	**14.194**		
**Total**	591.895	39			

## Discussion

The results of this study showed force decay in both elastic chain and coil spring groups over time, consistent with the results of previous studies.^3-6,11,12^

In all the groups, force decay occurred until the end of 3 weeks of experiment. However, a slight increase in force production occurred for the coil springs in the control group (between 1-hour and 24-hour, and 1-hour and 1-week intervals) and in the Orthokin group (between 1-hour and 24-hour intervals); though it was not statistically significant. This slight increase in force production can be attributed to some technical errors or even to the nature of NiTi coil springs. Some other studies have also reported this slight increase in force production during some specific periods of time.^12,13,22^

Considering the percentage of force degradation in each medium in the elastic chain group after 3 weeks of examination period (3), the highest percentage of force decay occurred with Persica mouthwash, which was not statistically significant compared to that in the control group. A notable result was seen in Orthokin mouthwash group, which showed the least force decay after 3 weeks, even in comparison with the control group. As mentioned previously, Orthokin mouth rinse contains sodium fluoride, chlorhexidine digluconate and zinc acetate. The effects of sodium fluoride mouthwash and chlorhexidine mouth rinse on the force decay of elastomeric chains were studied by Ramazanzadeh et al,^16^ who showed no statistically significant differences between force decay of elastic chains in saliva and in saliva + NaF solution,and by Al-Jumaili et al, who reported higher force degradation of elastic chains and NiTi coil springs in a mouthwash solution (chlorhexidine digluconate and sodium fluoride) in comparison with those in artificial saliva.^11^ In the present study, the force decay of Orthokin mouth rinse was lower than that in other test groups and even the control group, which might be attributed to the presence of zinc acetate in this mouth rinse, with a possible specific effect on the structure of the elastomeric chain. Therefore, further biochemical studies are suggested in future to help understand the effect of this probable reaction.

According to Tables 4 and 5, no statistically sig-nificant differences were detected in force degrada-tion after 3 weeks in the coil spring group.

The effect of different mouthwashes on the force decay of orthodontic traction aids have been reported previously. Pithon et al studied the effect of a bleaching agent-containing mouthwash on the force degradation of elastic chains and showed that the bleaching agent had no effect on force decay of elastomeric chains.^17^ Larrabee et al^3^ reported higher force decay in elastic chains exposed to a commercial mouth rinse containing alcohol compared to those exposed to water. Mahajan et al^24^ evaluated the effect of alcohol and alcohol-free mouth rinses on force decay of elastic chains, NiTi coil springs, and stainless steel coil springs. They concluded that the force decay of these groups in the alcohol-containing mouthwash was more than that in the alcohol-free mouthwash.^24^

Kumar et al^25^ studied the effect of Coca-Cola, tea and listerine mouthwash on the force degradation of elastic chains and showed the highest force degradation in the tea group and the lowest one in the Coca-Cola group compared to the control group.

To the best of the authors’ knowledge, no study has been conducted on the effect of Persica (a native herbal mouthwash) and Sensikin (an anti-hypersensitivity mouthwash) on the force degradation of elastic chains and NiTi coil springs to date. Likewise, no comparisons have been made between these mouth rinses. A study evaluated the effect of Orthokin mouth rinse on the tensile strength of elastomeric chains, concluding that after 28 days of experiment, there were no statistically significant differences in the tensile strengths of elastomeric chains in saliva, Orthokin, and Oral B solutions.^15^

One limitation of the present study was that the samples were removed from the pins 5 times for force measurements, which resulted in their drying due to the lack of saliva during the measurement periods. However, since this condition was identical in all the groups, it might have had minimum effects on the results. In the current study, the forces were measured by a digital force gauge; however, for more precise measurements, employing more accurate, but more expensive, instruments such as a universal testing machine is recommended for future studies.

## Conclusions

1. Elastomeric chains showed force degradation over time in all the experimental groups.

2. After 3 weeks, in the elastic chain group the least force decay was seen in the Orthokin mouthwash group.

3. After 3 weeks, in coil spring group there was no statistically significant difference in the percentage of force degradation between the groups.

4. It seems that orthodontists can prescribe these three mouth rinses to their patients with no concerns about increasing the force degradation of elastic chains and NiTi coil springs.

## Acknowledgments

The authors thank Shiraz Orthodontic Research Center for supporting this research (Grant #7232). This manuscript is based on a thesis by Dr Zahra Javanmardi. The authors also thank Dr Mehrdad Vossoughi of the Dental Research Development Center, of the School of Dentistry for the statistical analysis and Dr Shahram Hamedaniyan for improving the use of English in the manuscript and Dental Biomaterial Reaserch Center.

## Authors’ contributions

ZJ performed the study concept, literature review, data acquisition, experimental studies, data analysis, manuscript preparation and manuscript review.

PS performed the study design, definition of intellectual content, statistical analysis, manuscript preparation and manuscript editing.

## Funding

This study was financially supported by Shiraz Orthodontic Research Center (Grant#7232).

## Competing interests

The authors declare that they have no competing interests with regards to authorship and/or publication of this article.

## Ethics approval

Not applicable.

## References

[1] Halimi A, Azeroual MF, Doukkali A, El Mabrouk K, Zaoui F (2013). Elastomeric chain force decay in artificial saliva: An in vitro study. Int Orthod.

[2] Baty DL, Storie DJ, von Fraunhofer JA (1994). Synthetic elastomeric chains: A literature review. Am J Orthod Dentofacial Orthop.

[3] Larrabee TM, Liu SS, Torres-Gorena A, Soto-Rojas A, Eckert GJ, Stewart KT (2012). The effects of varying alcohol concentrations commonly found in mouth rinses on the force decay of elastomeric chain. Angle Orthod.

[4] Mirhashemi A, Saffarshahroudi A, Sodagar A, Atai M (2012). Force-degradation pattern of six different orthodontic elastomeric chains. J Dent (Tehran).

[5] Kochenborger C, Lopes da Saliva D, Marchioro EM, Vargas DA, Hahn L (2011). Assessment of force decay in orthodontic elastomeric chains: an in vitro study. Dental Press J Orthod.

[6] Weissheimer A, Locks A, de Menezes LM, Borgatto AF, Derech CD (2013). In vitro evaluation of force degradation of elastomeric chains used in orthodontics. Dental Press J Orthod.

[7] Eliades T, Eliades G, Silikas N, Watts DC (2005). In vitro degradation of polyurethane orthodontic elastomeric modules. J Oral Rehabil.

[8] Eliades T, Gioka C, Zinelis S, Makou M (2003). Study of stress relaxation of orthodontic elastomers; pilot method report with continuous data collection in real time. Hel Orthod Rev.

[9] De Genova DC, McInnes-Ledoux P, Weinberg R, Shaye R (1985). Force degradation of orthodontic elastomeric chains--a product comparison study. Am J Orthod.

[10] Ferriter JP, Meyers CE Jr, Lorton L (1990). The effect of hydrogen ion concentration on the force-degradation rate of orthodontic polyurethane chain elastics. Am J Orthod Dentofacial Orthop.

[11] Al-Jumaili KA, Ali AI (2011). Evaluation and Comparison of the effect of artificial saliva and Mouthwash Solution on Force Degradation of Different Types of Orthodontic Traction Aids (Comparative in Vitro Study). Al-Rafidain Dent J.

[12] Santos AC, Tortamano A, Naccarato SR, Dominguez-Rodriguez GC, Vigorito JW (2007). An in vitro comparison of the force decay generated by different commercially available elastomeric chains and NiTi closed coil springs. Braz Oral Res.

[13] Pires BU, Souza RE, Filho MV, Degan VV, Santos JCB, Tubel CAM (2011). Force degradation of different elastomeric chains and nickel titanium closed springs. Braz J Oral Sci.

[14] Nattrass C, Ireland AJ, Sherriff M (1998). The effect of environmental factors on elastomeric chain and nickel titanium coil springs. Eur J Orthod.

[15] Jamilian A, Sheibaniniya A, Kamali Z, Mousavi K. Tensile strength of orthodontic elastomeric chains – In vitro. Orthodontic CYBER Journal 2011 Jan. Available from: http://orthocj.com/2011/01/tensile-strength-of-orthodontic-elastomeric-chains-in-vitro/.

[16] Ramazanzadeh BA, Jahanbin A, Hasanzadeh N, Eslami N (2009). Effect of sodium fluoride mouth rinse on elastic properties of elastomeric chains. J Clin Pediatr Dent.

[17] Pithon MM, Rodrigues AC, Sousa EL, Santos LP, Soares Ndos S (2013). Do mouthwashes with and without bleaching agents degrade the force of elastomeric chains?. Angle Orthod.

[18] Salehi P, Momeni Danaie Sh (2006). Comparison of the antibacterial effects of persica mouthwash with chlorhexidine on streptococcus mutans in orthodontic patients. DARU.

[19] Al-Otaibi M, Al-Harthy M, Gustafsson A, Johansson A, Claesson R, Angmar-Mansson B (2004). Subgingival plaque microbiota in Saudi Arabians after use of Miswak chewing stick and toothbrush. J Clin Periodontol.

[20] Darout IA, Albandar JM, Skaug N (2000). Periodontal status of adult Sudanese habitual users of Miswak chewing sticks or tooth brushes. Acta Odontol Scand.

[21] Kalessi AM, Pack AR, Thomson WM, Tomkins GR (2004). An invitro study of the plaque control efficacy of Persica: A commercially available herbal mouthwash containing extract of Salvadora Persica. Int Dent J.

[22] Angolkar PV, Arnold JV, Nanda RS, Duncanson MG (1992). Force degradation of closed coil springs: an in vitro evaluation. Am J Orthod Dentofacial Orthop.

[23] Samuels RH, Rudge SJ, Mair LH (1998). A clinical study of space closure with nickel-titanium closed coil springs and an elastic module. Am J Orthod Dentofacial Orthop.

[24] Mahajan V, Singla A, Negi A, Jaj S (2014). Influence of alcohol and alcohol-free mouth rinses on force degradation of different types of space closure auxiliaries used in sliding mechanics. J Ind Orthod Soc.

[25] Kumar K, Shetty S, Krithika MJ, Cyriac B (2014). Effect of commonly used beverage, soft drink, and mouthwash on force delivered by elastomeric chain: a comparative in vitro study. J Int Oral Health.

